# Simulation Analyses of tDCS Montages for the Investigation of Dorsal and Ventral Pathways

**DOI:** 10.1038/s41598-019-47654-y

**Published:** 2019-08-21

**Authors:** Sagarika Bhattacharjee, Rajan Kashyap, Brenda Rapp, Kenichi Oishi, John E. Desmond, S. H. Annabel Chen

**Affiliations:** 1 Psychology, Nanyang Technological University, Singapore, Singapore; 2 Centre for Research and Development in Learning (CRADLE), Nanyang Technological University, Singapore, Singapore; 3 The Johns Hopkins University, Kreiger School of Arts and Sciences, Baltimore, United States; 4 The Johns Hopkins University, School of Medicine, Baltimore, United States; 5 Department of Neurology, The Johns Hopkins University, School of Medicine, Baltimore, United States; 6 Lee Kong Chian School of Medicine (LKC Medicine), Nanyang Technological University, Singapore, Singapore

**Keywords:** Human behaviour, Language, Reading

## Abstract

Modulating higher cognitive functions like reading with transcranial direct current stimulation (tDCS) can be challenging as reading involves regions in the dorsal and ventral cortical areas that lie in close proximity. If the two pathways are stimulated simultaneously, the function of dorsal pathway (predominantly used for graphophonological conversion) might interfere with the function of the ventral pathway (used for semantics), and vice-versa. To achieve functional specificity in tDCS for investigating the two pathways of reading, it is important to stimulate each pathway per session such that the spread of current across the cortical areas due to the two montages has minimal overlap. The present study intends to achieve this by introducing a systematic approach for tDCS analysis. We employed the COMETS2 software to simulate 10 montage configurations (5 for each pathway) for three electrode sizes: 5 × 5, 3 × 3, and 5 × 7 cm^2^. This diversity in montage configuration is chosen since previous studies found the position and the size of anode and cathode to play an important role. The values of the magnitude of current density (MCD) obtained from the configuration were used to calculate: (i) average MCD in each cortical lobe, (ii) number of overlapping coordinates, and (iii) cortical areas with high MCD. The measures (i) and (iii) ascertained the current spread by each montage within a cortical lobe, and (ii) verified the overlap of the spread of current between a pair of montages. The analyses show that a montage using the electrode size of 5 × 5 cm^2^ with the anode at CP5 and cathode at CZ, and another with anode at TP7 and cathode at nape of the neck are optimal choices for dorsal and ventral pathways, respectively. To verify, we cross-validated the results with ROAST. This systematic approach was helpful in reducing the ambiguity of montage selection prior to conducting a tDCS study.

## Introduction

Transcranial direct current stimulation (tDCS) is a non-invasive brain stimulation technique involving a pair of electrodes that are placed over the scalp in order to pass a low intensity current through the cortex^1–6^. Passage of this current through the underlying cortical areas causes depolarization and hyperpolarization of resting membrane potentials^7,8^. Such neurophysiological changes in the cortex induced by tDCS application can result in behavioural changes^7,8^. For example, cognitive behavioural changes in healthy individuals have been seen during decision- making^9,10^, learning^11,12^, attention^13^ and language^14^. In previous studies using tDCS, enhancement of higher cognitive functions like reading performance in both healthy individuals^15–18^ and patients with impaired reading have been found^19–21^. All these studies employed different configurations (size and position) of anode and cathode, and hence there has been little uniformity in tDCS montage applied to reading. Thus, the generalizability of the effectiveness of tDCS applied to reading remains uncertain due to a paucity of research on optimal montage selection.

**Figure 1:**
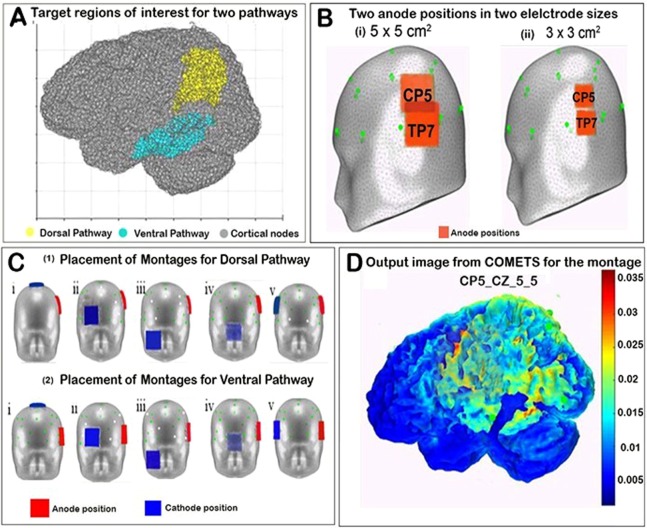
(**A**) The location of the total number of nodes in Talairach space (grey color). The target region of interest for dorsal (supramarginal gyrus) and ventral pathway (middle/inferior temporal gyrus) are shown in yellow and cyan color dots, respectively. (**B**) The position of anode (orange square patches) at CP5 for dorsal pathway montages and at TP7 for ventral pathway montages for electrode for two electrode sizes 5 × 5 cm^2^ (i) and 3 × 3 cm^2^ in left lateral view of head model of COMETS (ii). Note in (i) that the two square patches of tDCS at anode positions CP5 and TP7 are overlapping with each other. The green dots over the head model are the 10–20 electroencephalography electrode positions. (**C**) The position of anode (red) and cathode (blue) on the head model for electrode size 5 × 5 cm^2^ for dorsal (1) and ventral (2) pathways, respectively. (1) The position of anode is at CP5 and cathode is at (i) Cz, (ii) SO, (iii) contralateral maxilla, (iv) nape of the neck, and (v) contralateral homologous area CP4. (2) The anode (red) is placed at TP7 and the positions of the cathode (blue) were placed in the 5 locations as described in (1). (**D**) The COMETS output image showing the MCD distribution for a selected montage.

The reading network is thought to involve two neural pathways (dorsal and ventral). The dorsal pathway comprising the superior temporal gyrus (STG), temporal-parietal angular gyrus (AG), supramarginal gyrus (SMG), and Inferior frontal gyrus (IFG) is involved in grapheme to phonology conversion (sublexical). The ventral pathway consisting of the fusiform gyrus (FFG) to middle/inferior temporal gyrus (MTG) is involved in lexico-semantic functions associated with reading^22,23^ (see Fig. 1A). Thus, to specifically target either pathway independently, the tDCS montages will need to be configured to maximize selectivity to the cortical regions in the respective pathways. However, due to the close vicinity of the regions involved in the pathways, a key concern is whether a montage for the dorsal pathway might also depolarise the regions in the ventral pathway and vice versa. Thus, it is important to identify montages with the least overlap in the spatial spread of the electric field.

Recently, a meta-analysis by Westwood and Romani showed that the effect of tDCS on picture naming and word reading tasks were small, non-significant and variable^24^. We think that the probable reasons behind such insignificant findings in tDCS meta-analyses could be that the involved studies had variability in the (i) parameters of stimulation, (ii) target regions of interest and (iii) range of tasks under consideration. In this context, Bikson and Rahman, have suggested the need of anatomical and activity-related specificity in order to achieve tDCS related reliability and reproducibility^25^. To some extent, the present study is a step towards working on improving the reliability in tDCS experiments. Firstly, we outline montages that has been used in the past for stimulating reading behaviour only (activity- selective, see Table 1), thereby reflecting the inconsistency in montage selection. Finally, the present study also intends to achieve anatomical and functional specificity by targeting either supramarginal gyrus or middle/inferior temporal gyrus that are involved in two different subprocesses associated with reading (sublexical and lexical).

**Table 1:** The previous studies that investigated the effect of tDCS on reading behaviour.

Paper	Anode Position	Cathode Position	Electrode Size (cm^2^)	Current Intensity (mA)	N	Time (min)	Task Used
***Reading Studies On Healthy Individuals***							
Turkletaub *et al*.^18^	between T7 and TP7	between T8 and TP8	25	1.5	25	20	WRMT and TOWRE
Significant effect for TOWRE sight efficiency word was found whereas no effect was seen for Phonetic Decoding Efficiency and nonword accuracy.							
Thompson *et al*.^17^	Left CP5 Right CP6	Contralateral Mastoid	35	2	39	20	Phonological awareness, and TOWRE
Significant difference in TOWRE (Test of Word Reading Efficiency) sight word accuracy for left Vs right hemispheric stimulation. No significant difference in phoneme decoding and motor response was found. There was also a significant effect of spoonerism reaction time for anodal stimulation at right hemisphere.							
Flöel *et al*.^15^	CP5	Contralateral supraorbital (SO)	35	1	19	20	vocabulary learning task
Significant effect in anodal stimulation for accuracy data, lexical knowledge but no significant effect for reaction time was found.							
Forgione^61^	Between T7 & TP7,	between T8 and TP8	25	1.5	28	18	Word and non- word reading task
Accuracy and reaction time was significantly better for long words compared to short words. Right cathodal stimulation also enhanced the word and nonword reading speed.							
Sparing *et al*.^16^	CP5	SO And Cz	35	2	15	20	Picture naming task*
Significant difference in reaction time was obtained immediately after stimulation but not after 5 or 10 minutes of stimulation. Reference position at Cz was found to be better compared to SO position for an anode placed at CP5.							
Younger *et al*.^62^	Either on P3 or CP4	Contralateral SO	25	1.5	36	20	Single word reading, Rhyme judgement task
Participants who received anodal tDCS over left IPL were significantly better at reading efficiency relative to sham. They didn’t show significant difference for rhyme judgment relative to tDCS anodal stimulation over right IPL, and sham.							
Xue *et al*.^63^	Exp: Between T3 & P3	four return electrodes, CP5, CP1, Pz, and PO7	4 × 1	1.5	48	2O	Assembled and addressed phonology task
	Control: Oz	four return electrodes were PO3, O1, PO4, and O2,					
Left temporo parietal cortex stimulation specifically enhanced assembled phonology for trained word but no effect of stimulation on untrained word.							
Price *et al*.^64^	Central anode CP5	four cathode electrodes at C3, T7, P7, and P3	4 × 1	2.	18	20	Word pair task, Letter string task
Significant difference was observed for reaction time of meaningful word pairs by anodal stimulation. There was no significant difference in accuracy.							
***Reading studies on dyslexic individuals***							
Paper	Anode Position	Cathode Position	Electrode Size (cm^2^)	Current Intensity (mA)	N	Time	Task Used
Costanzo *et al*.^19^	between P7 and TP7	between P8 and TP8	25	1	19	20	Reading task
Significant difference for low frequency word accuracy and nonword speed was observed immediately after stimulation treatment. However, there was no significant effect for accuracy and speed in text and high frequency words.							
Costanzo *et al*.^20^	between P7 and TP7	between P8 and TP8	25	1	18	20	Lexical decision, phoneme blending, Verbal fluency and rapid naming.
Significant difference was seen post anodal stimulation for text reading error, lexical decision, and phoneme blending accuracy and reaction times. No significant effect in rapid naming task was observed							
Heth *et al*.^21^	V5	Right SO	Anode: 25 Cathode: 35	1.5	19	20	Rapid automatized naming

No significant effect in reading speed and error was seen immediately after stimulation but significant difference between groups was observed 1 week after stimulation.

(N = number of participants).*We included the paper in spite of using picture naming task as outcome measure because they targeted the anatomical region CP5 which is the area of interest of the present paper. So it is not task specific but partially serving the selection criteria by being anatomy specific.

In this respect, electrode position and size play an important role in stimulating the targeted cortical regions of interest. Studies have reported that the orientation of the current flow and the current density (defined as the amount of current per unit area) are influenced by the placement of the cathode^26,27^. Similarly, behavioural modulation is observed based on inter-electrode distance^28^. Reducing the size of the electrode has been reported to increase the focality of cortical excitation^4^. Although approaches for evaluating the cumulative influence of these parameters in identifying an optimal montage are a matter of great interest, they are currently lacking in the literature.

To fill this gap, the present study provides a systematic post processing analysis of current distributions via simulation and describes a computational pipeline that allows the tuning of these parameters to identify an optimal montage that can be applied to modulate reading behaviour. It specifically aims to facilitate the montage selection process for selectively stimulating the two reading routes. We hope the systematic approach might be helpful in selecting appropriate tDCS montage for other cognitive behaviour.

## Methodology

### Placements of the montages

The positions of the anode and cathode play a critical role in the distribution of current across a targeted area of interest. The conventional 10–10 electroencephalogram (EEG) electrode positions system is used to define the locations of anodes and cathodes^29^. As reading is usually left lateralized in the majority of right-handers^30^, the anode and cathode placements in the present study are selected so as to stimulate left hemisphere regions.

#### Anode position

The anode positions of the tDCS montages is located based on previous tDCS studies of reading. For example, Flöel *et al*., Sparing *et al*. and Thomson *et al*. placed the anode at CP5^15–17^, whereas Turkeltaub *et al*. and Costanzo *et al*. positioned it near TP7^18–20^. EEG based studies have shown that CP5 and TP7 electrodes map to supramarginal and middle/inferior temporal gyrus respectively^31^. Supramarginal gyrus forms an important region in the dorsal pathway of reading and the middle/inferior temporal gyrus is a key region in the ventral pathway^22,23^. Hence in our simulation, it appears to be reasonable to use CP5 and TP7 as anode positions for montages for the dorsal and ventral pathways of reading, respectively (Fig. 1B).

#### Cathode position

Cathode position in relation to the anode position can make a tDCS montage unipolar or bipolar. While the anode is placed on the scalp for both unipolar and bipolar montages, the cathode is usually placed at extra-cephalic locations (contralateral maxilla, nape of the neck) for unipolar, and cephalic locations (midline CZ, supraorbital SO, and contralateral homologous area) for bipolar^3^. With anode at CP5, cathode positions that are most used in reading are CZ and SO^15–17^. Similarly, with anode at TP7, a cathode position at contralateral homologous TP8 is conventionally used^18–20^. The montages and the parameters used in previous studies that investigated the effect of tDCS on reading are described in Table 1. These montages indicate a bias towards the bipolar arrangement. Furthermore, studies have found extracephalic cathode positions create focal distribution of current^32–34^. The hypothesis that extracephalic electrodes affecting physiological parameters^35^ has been contradicted by many studies^32,36–39^. Therefore, in addition to existing montages, hypothetical montages were introduced that included both cephalic and extra-cephalic cathode locations. This broadens our choices for identifying the best-fit montage for the two pathways of reading. In total, 10 montages (3 conventional and 7 hypothetical) were examined in the present study with 5 montages each for dorsal and ventral pathways. Cathode and anode placements for all the simulated montages for dorsal and ventral pathways can be visualised in Fig. 1C, respectively. These montages were further explored by varying the electrode sizes from (A) 5 × 5 cm^2^, to (B) 3 × 3 cm^2^ and (C) 5 × 7 cm^2^ (in the inferior-superior and anterior-posterior dimensions).

### Montage simulation

**Figure 2:**
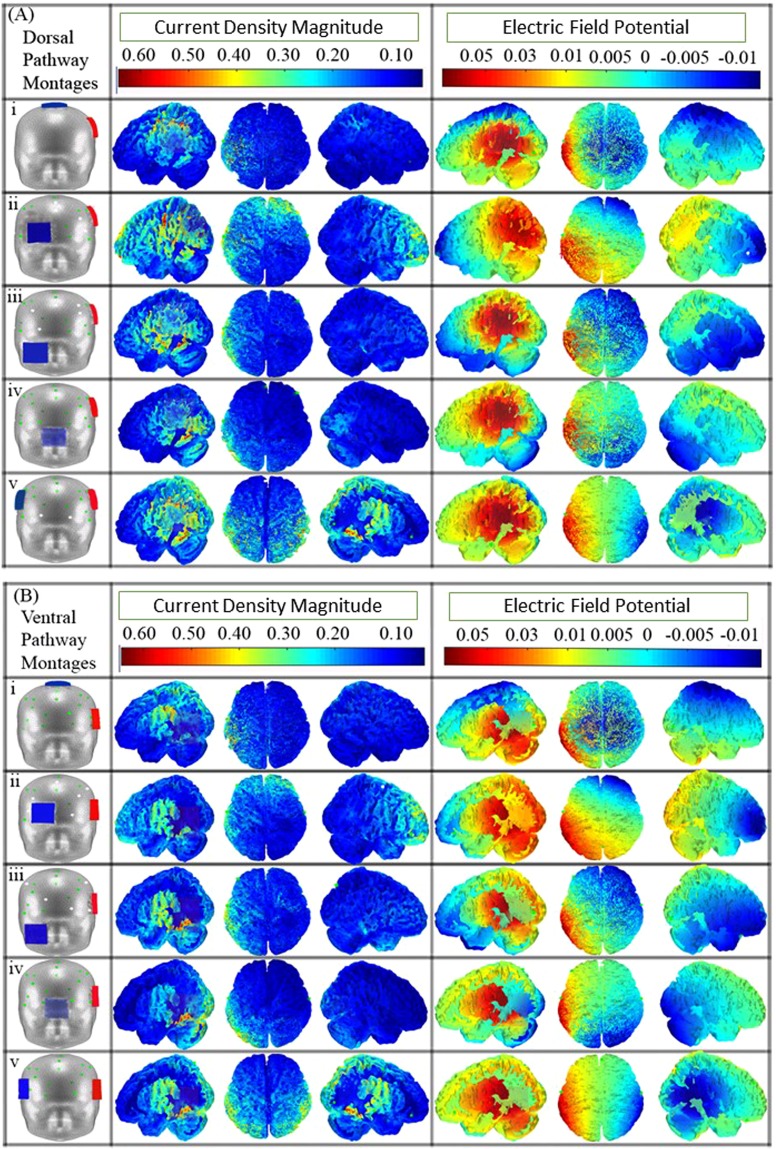
(**A**) The 5 dorsal pathway montages used in the study on anterior-posterior view of the head model of COMETS. The first column shows the position of anode (red) and cathode (blue) on the head model for electrode size 5 × 5 cm^2^. The position of anode is at CP5 and cathode is at (i) Cz, (ii) SO, (iii) contralateral maxilla, (iv) nape of the neck, and (v) contralateral homologous area CP4. The second column shows the distribution of the MCD (magnitude of current density) across the cortex. The third column shows the distribution of the electric field potential. All the montages shows the ‘current source’ (areas of high potential marked in red) at the left hemisphere. The ‘current sink’ (areas of low potential marked in blue) were formed at (i) the vertex for cathode at CZ, (ii) anterior pole for cathode at SO, (iii) anterior-inferior cortex for cathode at maxilla, (iv) posterior-inferior cortex for cathode at nape of the neck, and (v) right hemisphere for cathode at contralateral homologous area. (**B**) Montages, MCD distributions, and electric field distributions for the 5 ventral pathway montages for electrode size 5 × 5 cm^2^. The anode (red) is placed at TP7 and the positions of the cathode (blue) were placed in the 5 locations as described in (**A**).

The montages examined in the present study were simulated in COMETS2 (http://cone.hanyang.ac.kr/BioEST/Kor/Comets.html) which is a MATLAB based tDCS toolbox^33^. COMETS2 evaluates the magnitude of current density distributed across the 35,057 cortical nodes derived from a built-in head model using finite element method^33,34^. Magnitude of current density computed at each node is the norm of the current density values in the x-y-z directions^33^, and is a commonly used parameter in modelling studies^40^. To run the simulations of the montages in COMETS2, the built-in head model was imported and then the electrode position, size and current intensity were specified in a graphical user-based interface. The standard current intensity of 2 mA was considered, as this is a limit that is commonly observed^41,42^. On completion of a montage simulation (refer Fig. 2), two outputs of interest namely the *XYZ coordinates matrix* (35057 × 3) reflecting the location of cortical nodes (consists of x, y and z coordinates) in native headspace and the corresponding *magnitude of current density* (MCD) matrix (35057 × 1), were obtained. The native headspace matrix was mapped in Talairach space using the Fieldtrip toolbox (http://www.fieldtriptoolbox.org/)^43^. Anatomical locations of XYZ coordinates (33857 × 3) mapped to Talairach space were identified using Talairach client (http://talairach.org/client.html)^44,45^. The coordinates are plotted in Talairach space as shown in Fig. 1A.

### Parameters guiding montage selection

The cortical nodes (XYZ coordinates) that are mapped to Talairach space and their corresponding MCD values were used to calculate (i) *Average MCD per lobe* for each montage, (ii) *Number of overlapping coordinates* between a dorsal and ventral route montage, and (iii) *Cortical regions with high MCD* for each montage. The systematic approach that forms the basis for our montage selection is described in the subsequent sections.

#### Average MCD per lobe (for each montage)

Talairach client enables division of cortical nodes into left and right hemispheres with each hemisphere subdivided into frontal, parietal, temporal, occipital, limbic and sublobar lobes. The mean and standard deviations of MCDs were obtained for each lobe. Following this, the cortical lobe with maximum mean MCD value (max_MCD) and the cortical lobes with mean MCD values higher than the average MCD value across all lobes (avg_MCD) were identified. These cortical lobes identified from these values (max_MCD and avg_MCD) lays the foundation for selecting an appropriate montage and will be referred to as “lobe selectivity configuration analysis”. We will further discuss the lobe selectivity configuration analysis in section 2.4 (Montage selection).

#### Number of overlapping coordinates (for each montage pair)

In order to evaluate the extent of overlap between dorsal and ventral pathway montages, a thresholding of MCD values at 50% of maximum MCD was performed for each montage separately. The choice of threshold was based on our observation that at a particular threshold the MCD distribution (obtained as output of a montage simulation in COMETS2) over a cortical area of interest was substantial. For example, for a montage simulated in COMETS2, we can see the MCD distribution (Fig. 1D) for coordinates across the cortex with MCD values > 0.015, which is 50% of the maximum MCD value. On this basis, the number of above-threshold coordinates common for each pair of montages was calculated.

#### Cortical regions with high MCD (for each montage)

The goal of the present parameter is to identify the cortical nodes that are maximally stimulated and their corresponding anatomical locations. At this point, it is worth recalling that each of the 35,057 cortical nodes has a particular MCD value. We selected those nodes that are above the 50% threshold of maximum MCD values (as described above). The anatomical locations of these nodes were identified in Talairach space. To identify if a region of interest (for example, supramarginal gyrus) was maximally stimulated, we clustered the cortical nodes according to the gyri, and total MCD of each cluster (CMCD) was calculated. In this way, for each montage, Cn clusters (where n > 0) were formed.

### Montage selection

We used the parameters above to help select the optimal montage that targets the specific dorsal and ventral pathways of reading based on three guiding principles. The 1^st^ principle helps to select the most appropriate montage for each pathway, and the 2^nd^ principle helps to select the best pair of montages for stimulating both the pathways. The 3rd principle helps verify the appropriateness of the candidate montages derived from applying the 1^st^ and 2^nd^ principles. These three guiding principles for montage selection were derived based on the pathways to be investigated. Thus, different research questions may have different ways of applying these parameters.

**Figure 3:**
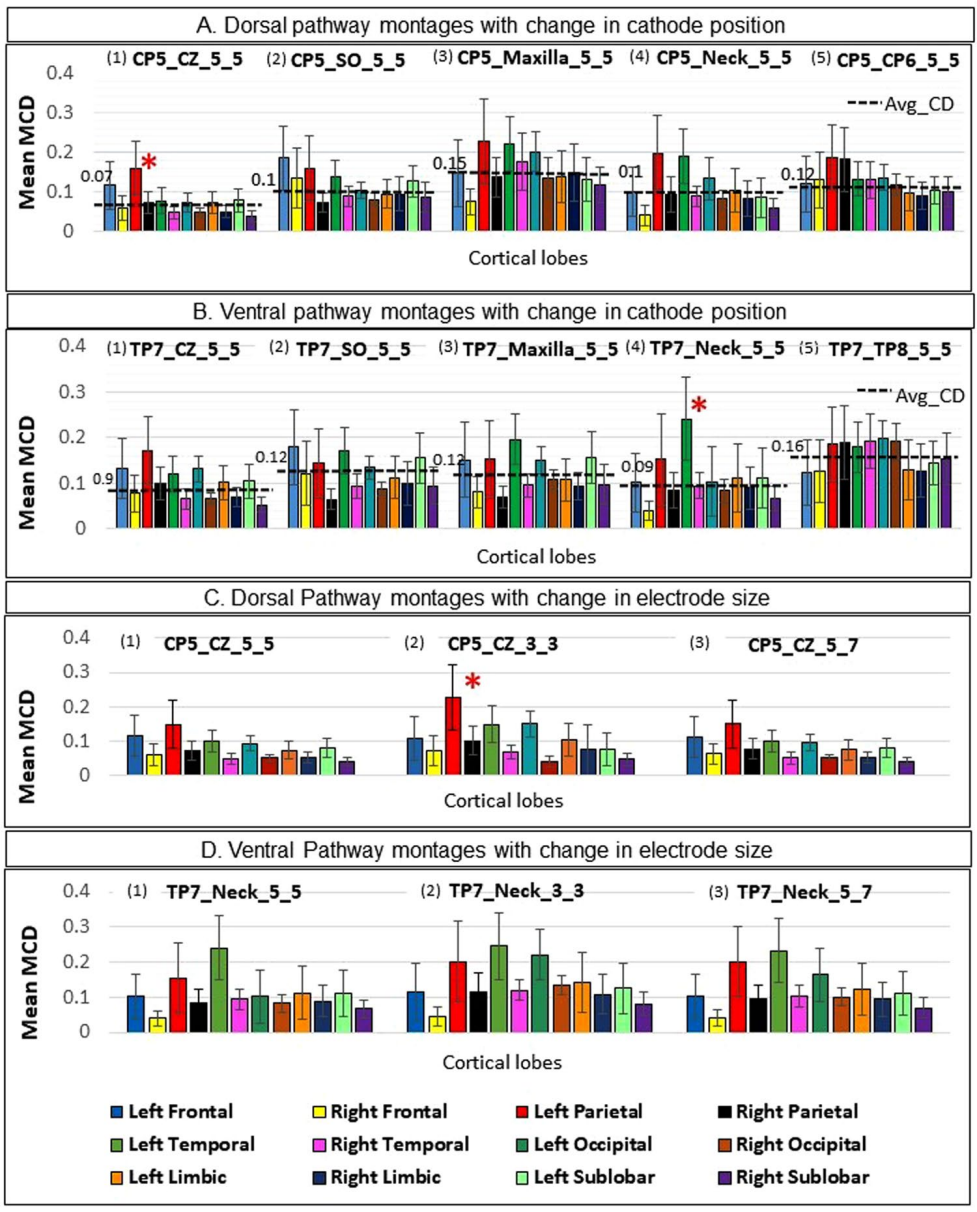
(**A**,**B**) The lobe selectivity configuration analysis for dorsal (anode at CP5) and ventral (anode at TP7) route montages, respectively. The cathode was placed at (i) CZ, (ii) SO, (iii) Maxilla, (iv) Nape of Neck, (v) contralateral homologous areas. The montage CP5_CZ_5_5 and TP7_Neck_5_5 shows max_MCD (magnitude of current density) at left parietal lobe and temporal lobe (demarcated by *), respectively with least number of lobes crossing the avg_MCD line (−). (**C**,**D**) Shows the lobe selectivity configuration analysis for dorsal (CP5_CZ) and ventral (TP7_Neck) pathway montages for three electrode sizes (i) 5 × 5 cm^2^, (ii) 3 × 3 cm^2^, (iii) 5 × 7 cm^2^. For CP5_CZ, there is significant difference in the max_MCD values seen at left parietal lobe (Red bar) with change in electrode size from (i) 5 × 5 cm^2^, to (ii) 3 × 3 cm^2^ (p < 0.01) (demarcated by *).

1. The first guiding principle for montage selection is the Lobe selectivity configuration analysis that includes the MCD map for each of the cortical lobes. The montage that showed max_MCD at either of the two targeted cortical areas namely the left parietal lobe and temporal lobe passed the first level of screening for their appropriateness as montages for dorsal or ventral pathway stimulation, respectively. Given that multiple montages exhibited max_MCD at the desired cortical lobe for each pathway, a second level of screening was used based on minimal spread of MCD to the remaining lobes. Spread was determined by the count and extent to which the mean MCD values of the remaining lobes exceed the avg_MCD value (refer Fig. 3). Once a montage with minimal spread and focal stimulation was selected for each pathway, differences in the lobe selectivity configuration analysis for two electrode sizes namely 3 × 3 cm^2^, and 5 × 7 cm^2^ was computed.
  The idea behind the analysis of average CD per lobe is to obtain a montage that could result in the maximum intensity of the current in the cortical lobe of interest. It is said that bipolar montage has an advantage of achieving maximum intensity at the target region but compromises the focality^46^. The additional measure of lobe selectivity configuration analysis will facilitate the montage selection process by ensuring that there is least spread of current to other cortical lobes. Here, we recognise the fact that multi-electrode configuration is optimal for achieving the maximum focality but might compromise on intensity^46^. An optimal balance between intensity and least spread of current to other cortical regions are important for effective modulation of task performance by tDCS (especially in a case like reading).
2. The second guiding principle for selection of a pair of montages is the *Number of overlapping coordinates*. A dorsal and a ventral pathway montage constituted a pair for calculating the number of overlapping coordinates. All 25 possible combinations of 5 dorsal and 5 ventral pathway montages with electrode size 5 × 5 cm^2^ were evaluated (note that that this assessment was done independently of the result obtained from the 1^st^ assessment). The pair with least number of overlapping coordinates is considered to be the optimal choice. This will ensure that the present montage selection procedure will select the montage that can stimulate one target region of interest, by selectively excluding another region. Such an approach is beneficial for modulating those behaviours where two sub processes are involved (for example, sublexical and lexical in case of reading). And the research question in hand intends to tease apart these sub processes by two separate tasks. In that case, the desired differentiation in the task performances will not be visible, if cortical regions underlying the two sub processes are stimulated simultaneously.
  Additionally, since studies have reported that decrease in electrode size results into focal spread of current^4,41^, we hypothesised that decrease in electrode size should result in less overlap of MCD distribution for two adjacently placed montages (Fig. 1B-i,ii). We tested this hypothesis by varying the electrode sizes from 5 × 5 cm^2^ to 3 × 3 cm^2^, and 5 × 7 cm^2^ for the 25 dorsal and ventral route montage combinations.
3. CMCDs were calculated for two (dorsal and ventral) chosen montages based on the lobe selectivity configuration analysis and number of overlapping coordinates to confirm if high CMCDs occurred around the cortical regions of interest. This parameter investigates, whether the maximum intensity of current is formed in the specific gyrus within a cortical lobe. The 3^rd^ guiding principle is that, the clusters with high CMCDs were expected to be found in and around the supramarginal gyrus and the middle/inferior temporal gyrus for dorsal and ventral montages, respectively.

All the above three parameters can be applied either independently or in conjunction to select an appropriate montage. However, analysis made using all the three parameters should in principle facilitate robust montage selection. In order to check the robustness of the result, we performed a reanalysis for the montage selection process from the data obtained from another simulation pipeline called Realistic vOlumetric-Approach to Simulate Transcranial Electric Stimulation — ROAST^47^.

Finally, some additional analyses were also performed over the montages that were selected utilizing the above three principles. These analyses are independent of montage selection process rather perform a sensitivity analysis^48^, where the affect of variations in total current intensity (from 2 mA to 1 mA) and displacement of electrodes (within a range of 1 cm) over the lobe selectivity configuration analysis were evaluated (for details refer to supplementary). The matlab code for this framework can be downloaded from 10.21979/N9/DMWPZK.

## Results

### Montage overview

**Figure 4:**
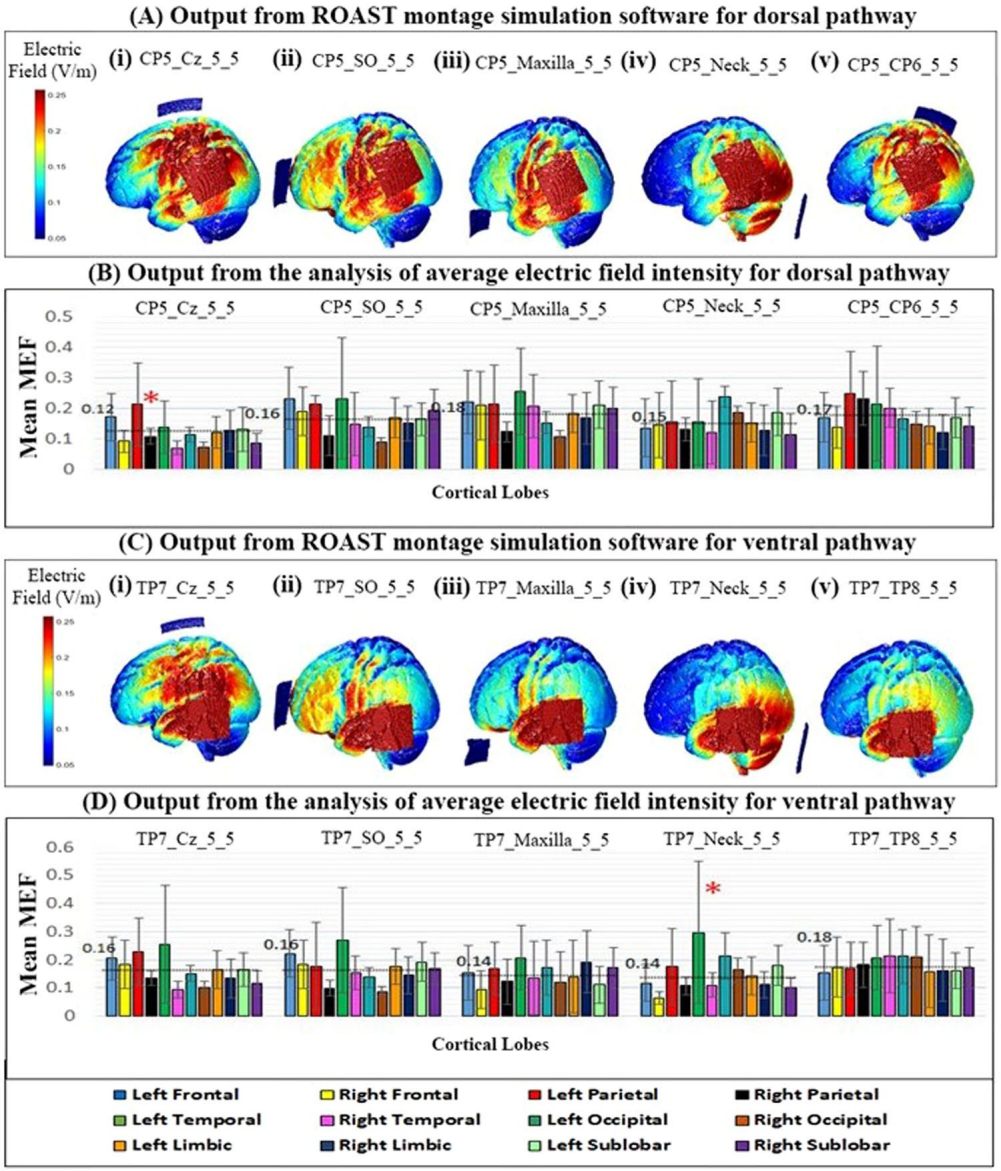
(**A**) The electric field intensity map of the simulated montages in the head model of ROAST for dorsal pathway with anode at CP5 and cathode at (i) CZ, (ii) SO, (iii) Maxilla, (iv) Nape of Neck, (v) Contralateral homologous areas. (**B**) The lobe selectivity configuration analysis with mean MEF (magnitude of electric field intensity) for dorsal pathway (anode at CP5). The cathode was placed at (i) CZ, (ii) SO, (iii) Maxilla, (iv) Nape of Neck, (v) contralateral homologous areas. The montage CP5_CZ_5_5 (i) shows max_MEF (magnitude of electric field) at left parietal lobe (demarcated by *) with least number of lobes crossing the avg_MEF line (−). (**C,D**) Same as (**A**) for ventral pathway montages. The montage with anode a TP7 and cathode at neck (iv) shows max_MEF at left temporal lobe (demarcated by *).

The 10 simulated montages (5 each for dorsal and ventral pathway) with placement of anode and cathode are shown in Fig. 2A, B, respectively. The second column of each figure shows the colour maps of the MCDs representing the areas stimulated by a montage. The third column showing electric field potential gives us an estimate of the flow of current across the brain. From the 3^rd^ column, it can be observed that all the montages had the ‘current source’ (areas of high potential marked in red) at the left hemisphere; however, the ‘current sink’ (areas of low potential marked in blue) varied based on the placement of reference electrode. The current sinks can be seen to be formed at (i) the vertex for cathode at CZ, (ii) anterior pole for cathode at SO, (iii) anterior-inferior cortex for cathode at maxilla, (iv) posterior-inferior cortex for cathode at nape of the neck, and (v) right hemisphere for cathode at contralateral homologous area. The electric field map for all the 10 montages in dorsal and ventral pathway obtained from ROAST are shown in Fig. 4(A,C).

### **Average MCD per lobe**

The results of average MCD per lobe for dorsal and ventral route montages can be organised in two categories namely (a) the effect of cathode position, and (b) the effect of electrode size in the format X_Y_L_B.

#### Effect of cathode position

Dorsal pathway: The average MCD per lobe was compared for the montages in the dorsal pathway with anode at CP5 and cathode at midline CZ (CP5_CZ_5_5), contralateral SO (CP5_SO_5_5), maxilla (CP5_Maxilla_5_5), nape of the neck (CP5_Neck_5_5), and contralateral homologous area CP6 (CP5_CP6_5_5) are shown in Fig. 3A. In all these montages, the mean MCDs in left hemispheric lobes are higher than the corresponding right hemispheric lobes.

Figure 3A(iii,iv) shows max_MCD at left parietal and temporal lobe for the montages CP5_Maxilla_5_5 (0.22 mA) and CP5_Neck_5_5 (0.19 mA). Similarly, for the montage CP5_CP6_5_5, the max_MCD (0.18 mA) is seen at both left and right parietal lobe (Fig. 3A(v)). This indicates that the montages CP5_Maxilla_5_5 and CP5_Neck_5_5 will comparably stimulate left parietal and temporal lobe; whereas the montage CP5_CP6_5_5 will equally stimulate left and right parietal lobe. This is clearly not desirable in the present context as focal current distribution to left parietal lobe is sought.

The max_MCD is seen at left parietal lobe for the montage CP5_CZ_5_5 (0.15 mA) and at left frontal lobe (0.18 mA) for the montage CP5_SO_5_5 (shown in Fig. 3A(i,ii), respectively). These two montages were commonly used in previous tDCS studies on reading^15–17^. For the montage CP5_CZ_5_5, the left parietal and the frontal lobe exceeds the avg_MCD by a margin of 100% and 53%, respectively. However for the montage CP5_SO_5_5, the cortical lobes that exceed the avg_MCD are left frontal lobe by 63%, left parietal lobe by 36%, left temporal lobe, left sublobar lobe, and right frontal lobe each by 18%. Moreover, ANOVA shows that the CD in each cortical lobe within the montage CP5_Cz_5_5 are significantly different from each other (F = 2,756.2; *P* < 0.001) and post-hoc analysis shows left parietal lobe to receive significantly higher amount of current compared to left frontal lobe (t = 25.17, *P* < 0.05). The lobe selectivity configuration analysis therefore indicates that the montage with anode at CP5 and cathode at CZ (CP5_CZ_5_5) stimulates the left parietal lobe with less diffusivity of current to other lobes.

Ventral pathway: Similarly, the average MCD per lobe was compared for the montages in the ventral pathway with anode at TP7 and cathode at midline CZ (TP7_CZ_5_5), contralateral SO (TP7_SO_5_5), maxilla (TP7_Maxilla_5_5), nape of the neck (TP7_Neck_5_5), and contralateral homologous area TP8 (TP7_TP8_5_5) depicted in Fig. 3B. A literature search indicated that the TP7_TP8_5_5 configuration was the only montage applied in previous reading studies for the ventral pathway^18–20^. Figure 3B(v) reflects approximately equivalent amount of MCD getting distributed to left and right parietal, temporal and occipital lobes for the montage TP7_TP8_5_5, which shows that both right and left hemisphere have equivalent current distribution. This configuration can be useful when the research question in hand requires the left hemispheric regions to be depolarised by the anode and the right hemispheric regions to be hyperpolarised by the cathode. However, if only depolarisation of left hemispheric region by the anode is desired with minimal effect on the right hemisphere, the TP7_TP8 montage might not be ideal. Therefore, TP7_TP8_5_5 is compared with all other hypothetical montages introduced in the present study (Fig. 3Bi–iv) to select a montage that satisfies the principle of focally targeting the left hemispheric regions in the ventral pathway.

For the montage TP7_CZ_5_5 and TP7_SO_5_5, the max_MCD is seen in the left parietal lobe (0. 16 mA) and left frontal lobe (0.17 mA) as in Fig. 3B(i) and in Fig. 3B(ii), respectively. Clearly, these cortical lobes are not the desired targets for the ventral pathway of reading. For the other two montages TP7_Maxilla_5_5 and TP7_Neck_5_5 (in Fig. 3Biii,iv), the max_MCD is seen in the left temporal lobe as 0.19 mA and 0.24 mA respectively, which is the desired region for the ventral pathway. For the montage TP7_Maxilla_5_5, the MCD value of cortical lobes that exceeds the avg_MCD value are at left frontal lobe and parietal lobes by 36.6%, left temporal lobe by 58.8%, left limbic lobe by 25% and left occipital by 16.6%. Whereas, the MCD value of cortical lobes that exceeds the avg_MCD value are at left temporal lobe by 118.1% and left parietal lobe by 36.3%, for the montage TP7_Neck_5_5. Moreover, ANOVA shows that the CD in each cortical lobe within the montage TP7_Neck_5_5 are significantly different from each other (F = 2,095.37; *P* < 0.001) and post hoc analysis shows left temporal lobe to receive significantly higher amount of current compared to left frontal lobe (t = 22.61; *P* < 0.05). Thus, the montage TP7_Neck_5_5 generates focal MCD distribution to left temporal lobe.

#### Effect of electrode size

The effect of different electrode sizes 3 × 3 cm^2^, 5 × 7 cm^2^ and 5 × 5 cm^2^ for dorsal pathway montage CP5_CZ_5_5 on max MCD value for left parietal lobe was compared using *one way ANOVA* as shown in Fig. 3C(i–iii). The analysis of variance shows a significant result as *F (2, 7863)* = (858.86), *p* < *0.001*. A Tukey post-hoc analysis shows a significant increase in max_MCD value for left parietal lobe for electrode size 3 × 3 cm^2^ (*p* < *0.001*); and no significant difference for electrode size 5 × 7 cm^2^ (*p* > *0.05*) compared to 5 × 5 cm^2^. However, there is no difference in lobe selectivity configuration analysis for the two electrode sizes.

For the ventral pathway, the effect of electrode sizes 3 × 3 cm^2^, 5 × 7 cm^2^ and 5 × 5 cm^2^ for the montage TP7_Neck_5_5 on the the max_MCD value at left temporal lobe was compared using a *one way ANOVA as* shown in Fig. 3D(i–iii). No significant effects were found (*F (2, 1320)* = (0.3992), *p* = 0.6710). This shows that a difference in electrode size does not change the pattern of distribution of MCD across the cortical lobes. However, decreasing the size of electrodes may increase the max_MCD value.

Similar results were obtained from the analysis of average magnitude of electric field intensity (MEF) for the data obtained from ROAST (shown in Fig. 4B,D). The norm values of MEF were used for current analysis. Like CD, Electric field intensity (E) is also a frequently used parameter in the past tDCS studies Electric field intensity (E) is also a frequently used parameter in the past tDCS studies where CD is related to E as in *j* = *σ*E, *where j* = *current density* (mA^2^), *E* = *Electric field intensity* (V/m) and *σ* = *resistivity of the tissue*. With the constant resistivity in cortex, J and E demonstrates equivalent distribution^40^.

For dorsal pathway, the montage CP5_CZ_5_5 obtained max_MEF (equivalent for max_MCD, described above) at left parietal lobe (0.21 V/m) with left frontal lobe crossing the avg_MEF (equivalent for avg_MCD, described above) by 41.6%, left temporal lobe by 8.3% and left limbic lobe by 0.8% (see, Fig. 4B). One way ANOVA shows MEFs in each cortical lobe within the montage CP5_Cz_5_5 are significantly different from each other (F = 6,147.55; *P* < 0.001). Post hoc analysis shows left parietal lobe to receive significantly higher amount of current compared to left frontal lobe (t = 30.5, *P* < 0.05) and left temporal lobe (t = 53.78; *P* < 0.05).

For ventral pathway the montage TP7_Neck_5_5 forms the max _MEF at left temporal lobe (0.3 V/m) with left parietal lobe crossing the avg_MEF by 14.2%, left occipital lobe by 42.8%, right occipital lobe by 7%, and left sublobar lobe by 21.4% (see, Fig. 4D). One way ANOVA shows MEFs in each cortical lobe within the montage TP7_Neck_5_5 are significantly different from each other (F = 6,679.73; *P* < 0.001). Post hoc analysis shows left temporal lobe to receive significantly higher amount of current compared to left parietal lobe (t = 13.20; *P* < 0.05) and left occipital lobe (t = 47.08, *P* < 0.05). This shows that the CP5_CZ_5_5 and TP7_Neck_5_5 are the winning montages following a reanalysis with the simulation data obtained from ROAST.

### Number of overlapping coordinates

As noted previously, the number of overlapping coordinates could be affected by (a) differences in cathode position, and/or (b) differences in electrode size.

#### Effect of cathode position

**Figure 5:**
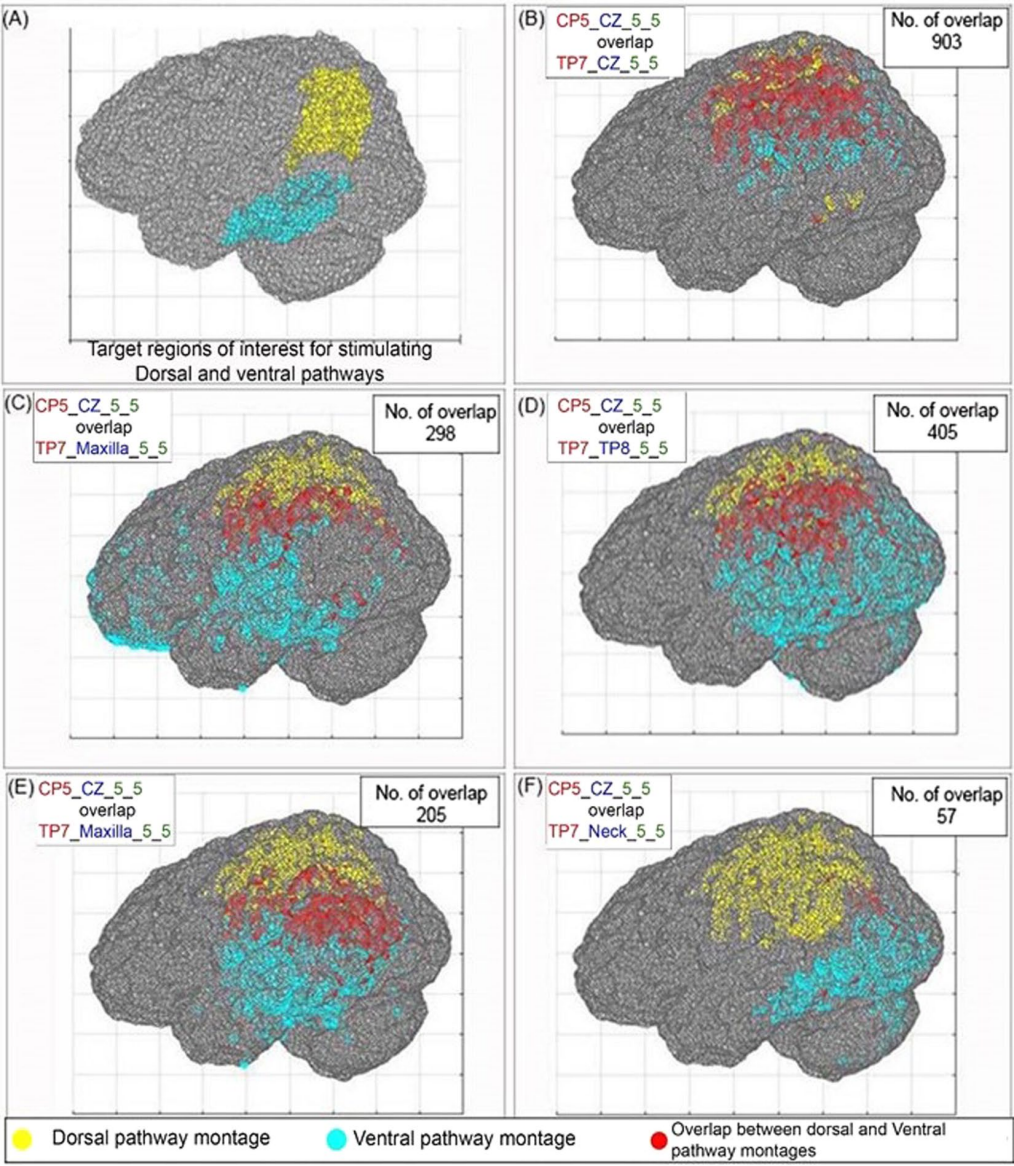
(**A**) The target regions of interest for dorsal and ventral pathways. (**B**) The highest number of overlapping coordinates is seen between CP5_CZ_5_5 and TP7_CZ_5_5. (**C,D**) Two poles of high MCD are formed for the ventral route montages TP7_SO_5_5 and TP7_TP8_5_5. (**E**) Moderate overlap is seen between CP5_CZ_5_5 and TP7_Maxilla_5_5. (**F**) Least overlap is seen between CP5_CZ_5_5 and TP7_Neck_5_5. The yellow and cyan color dots represent the coordinates that cross the 50% threshold limit for dorsal and ventral pathway montages. The red dots represent the overlap between these two montages.

The degree of overlap between the dorsal route montage CP5_CZ_5_5 and the 5 ventral route montages namely TP7_CZ_5_5, TP7_SO_5_5, TP7_TP8_5_5, TP7_Maxilla_5_5, and TP7_Neck_5_5 are depicted in Fig. 4B–F. We report the overlap from only these 5 pairs of montages because focal MCD distribution to left parietal lobe was obtained for the montage CP5_CZ_5_5 based on the assessment from *lobe selectivity configuration analysis*. Furthermore, this choice is also supported by the previous experiment of Sparing *et al*., where better results in terms of behavioural outcomes were obtained for the CP5_CZ_5_5 montage compared to CP5_SO_5_5^16^. Therefore, to find the montage pair with least overlap we opted to report the comparison of different ventral route montages in combination with the dorsal route montage CP5_CZ_5_5. We report the number of overlapping coordinates between the dorsal pathway montage CP5_CZ_5_5 and the 5 ventral pathway montages TP7_CZ_5_5, TP7_SO_5_5, TP7_TP8_5_5, TP7_Maxilla_5_5, and TP7_Neck_5_5 are 903, 298, 405, 205, and 57, respectively. As seen in Fig. 5F, CP5_CZ_5_5 and TP7_Neck_5_5 combination shows the least number of overlapping coordinates (57) among all other combinations. Although the numbers are different from COMETS2, analysis from ROAST shows that the same combination of CP5_CZ_5_5 and TP7_Neck_5_5 was found to have least number of overlapping coordinates (n = 75, Table 2).

**Table 2:** Number of overlapping coordinates for the combination of dorsal route montage CP5_Cz_5_5 with ventral route montages with anode at TP7 and cathode at (i) Cz (ii) SO, (iii) Maxilla, (iv) nape of the neck, (v) contralateral homologous area TP8, for electrode size 5 × 5 cm^2^.

Number	Dorsal Route Montage	Ventral Route Montage	No. of Overlapping coordinates (ROAST)	No. of Overlapping coordinates (COMETS2)
(i)	CP5_CZ	TP7_CZ	839	903
(ii)	CP5_CZ	TP7_SO	791	298
(iii)	CP5_CZ	TP7_Maxilla	725	405
(iv)	CP5_CZ	TP7_TP8	125	206
(v)	CP5_CZ	TP7_Neck	75	45

#### Effect of electrode size

**Figure 6:**
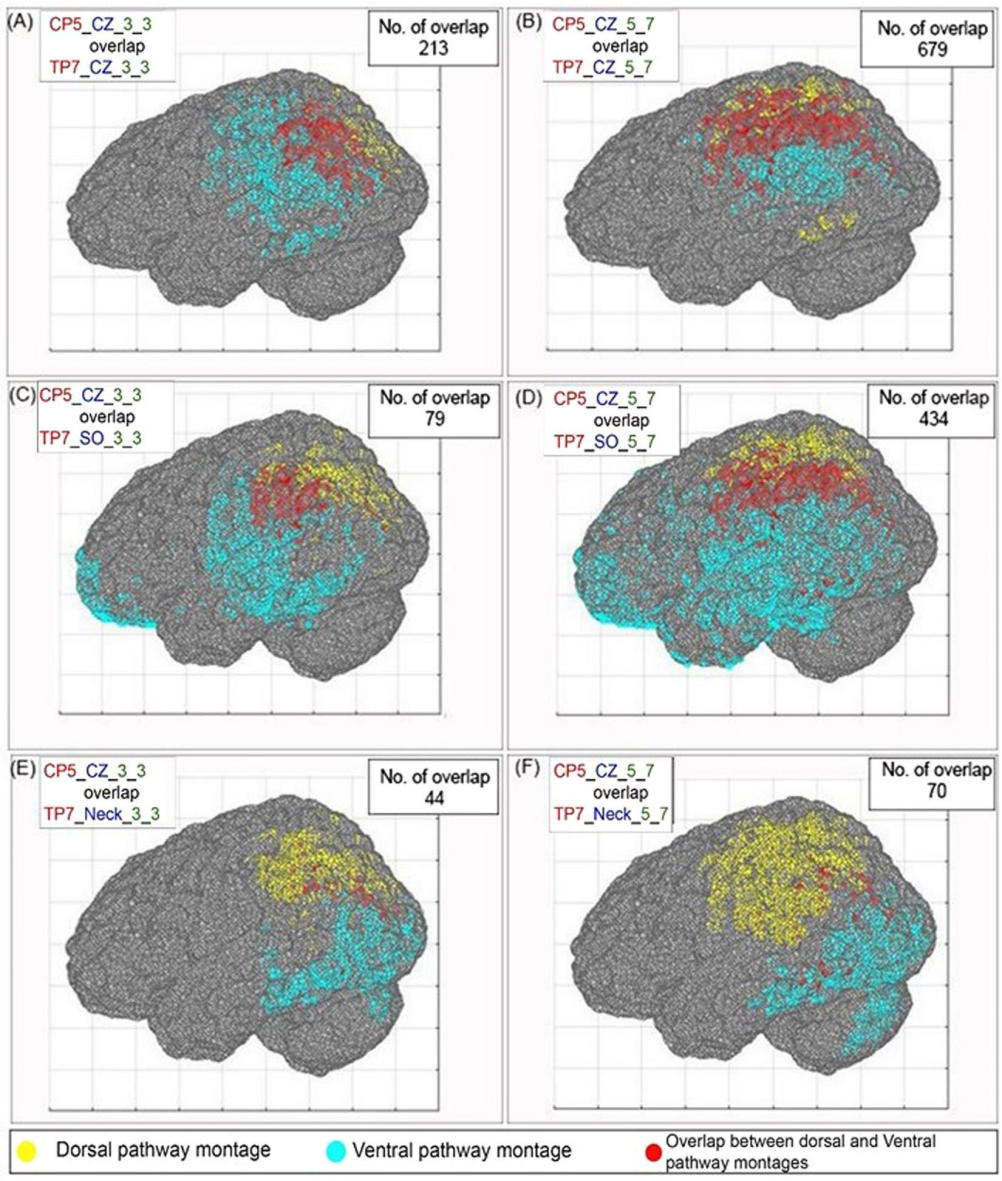
(**A,B**) The number of overlapping coordinates for the montages CP5_CZ_5_5 and TP7_CZ_5_5 for two electrode sizes 3 × 3 cm^2^ and 5 × 7 cm^2^. (**C,D**) The number of overlapping coordinates for the montages CP5_CZ_5_5 and TP7_SO_5_5 for two electrode sizes 3 × 3 cm^2^ and 5 × 7 cm^2^. (**E,F**) The number of overlapping coordinates for the montages CP5_CZ_5_5 and TP7_Neck_5_5 for two electrode sizes 3 × 3 cm^2^ and 5 × 7 cm^2^. The yellow and cyan color dots represent the coordinates that cross the 50% threshold limit for dorsal and ventral pathway montages. The red dots represent the overlap between these two montages.

The effect of electrode size on the number of overlapping coordinates is shown for three pairs of montages. These pairs consist of one dorsal route montage CP5_CZ and three ventral route montages TP7_CZ, TP7_SO, and TP7_Neck. These combinations were chosen to represent both the bipolar (TP7_CZ and TP7_SO) and unipolar varieties (TP7_Neck). The overlap for these montage pairs for electrode size 5 × 5 cm^2^ are shown in Fig. 5B,C,F; for 3 × 3 cm^2^ in Fig. 6A,C,E, and for 5 × 7 cm^2^ in Fig. 6B,D,F. One question of interest is whether a reduction in overlap between two montages that have the same reference electrode could be achieved by reducing the electrode size. In Figs 5 and 6, we do observe a reduction in the total number of overlapping coordinates with decreasing electrode size. However, it seems that this decrease is at least partly a consequence of the decrease in the total number of coordinates exceeding the 50% threshold, which tends to occur with decrease in electrode size. We will explain this with an example.

In order to compare the proportions of number of overlapping coordinates (*N overlap*) to the total number of above threshold coordinates (*N* > *thresh*) for any two pairs of montages pair1 and pair2, two proportions are defined as *p1* and *p2*, respectively. We define *p* as,

$$p=(N\,overlap)/(N > thresh),\,\forall \{(N\,overlap) > 0;\,(N > thresh) > 0\}:p > 0$$

Further, we define the ratio (*r*) for two values of *p* (*p1* and *p2*) as

$$r=|p1-p2|/(p1+p2),\,\forall p1 > 0;p2 > 0:0\le r\le 1$$

Higher values of *r* indicate that the difference between *p1* and *p2* is larger. We will now calculate the values of *p1, p2* and *r* by keeping the pair1 (CP5_CZ_5_5 & TP7_CZ_5_5) as constant and varying the pair2 (CP5_CZ & TP7_CZ) across two electrode sizes (A) 3 × 3 cm^2^ and (B) 5 × 7 cm^2^, and (c) changing the cathode position of one of the montage TP7_CZ from CZ to Neck while maintaining the electrode size of the pair as 5 × 5 cm^2^; as shown in Table 3.

**Table 3:** The values of *p1, p2* and *r* for the combinations A, B, and C.

A	Pair1	N overlap	N > thresh	p1	Pair2	N overlap	N > thresh	p2	*r value*
	CP5_CZ_5_5	903	1695	0.53	CP5_CZ_3_3	213	1092	0.19	0.46
	TP7_CZ_5_5				TP7_CZ_3_3				
B	Pair1	N overlap	N > thresh	p1	Pair2	N overlap	N > thresh	p2	*r value*
	CP5_CZ_5_5	903	1695	0.53	CP5_CZ_5_7	679	1465	0.46	0.07
	TP7_CZ_5_5				TP7_CZ_5_7				
C	Pair1	N overlap	N > thresh	p1	Pair2	N overlap	N > thresh	p2	*r value*
	CP5_CZ_5_5	903	1695	0.53	CP5_CZ_5_5	57	1397	0.04	0.86
	TP7_CZ_5_5				TP7_Neck_5_5				

N overlap = Number of overlapping coordinates, N > thresh = Number of coordinates above the threshold. *p1* and *p2* = proportion of N overlap/N > thresh for pair 1 and Pair 2 montages, and $\,r=|p1-p2|/(p1+p2)$.

The value of *r* calculated for the pair of montage CP5_CZ_5_5 & TP7_CZ_5_5 by varying the electrode sizes from 5 × 5 cm^2^ to (1) 3 × 3 cm^2^ (combination A) and (2) 5 × 7 cm^2^ (combination B) are 0.46 and 0.07, respectively. These values of *r* (*r* ≪ 1) suggests that the difference between the two proportions *p1* and *p2* is minimum, thereby implying that the proportion of *N overlap* to *N* > *thresh* is remaining fairly constant with change in electrode size. In contrast, the value of r for the combination C with change in cathode position is 0.86. The higher value of *r* (*r* → 1) implies that the decrease in *N overlap* is relatively independent of the decrease in *N* > *thresh*. This indicates that the position of the cathode is playing a larger role compared to electrode size for determining the pair of montage with least overlap (i.e. CP5_CZ_5_5 and TP7_Neck_5_5). Moreover, it is also visually evident from Figs 4 and 5 that the electrode sizes that resulted in focal spread of current did not result in better separation of above-threshold coordinates in space, as one might have expected. This may be due to the fact that the electric field in tDCS is generated at locations slightly outside of the cortical area beneath the anode and tends to spread toward the direction of the cathode^49–51^.

### **Cortical regions with high MCD**

**Figure 7:**
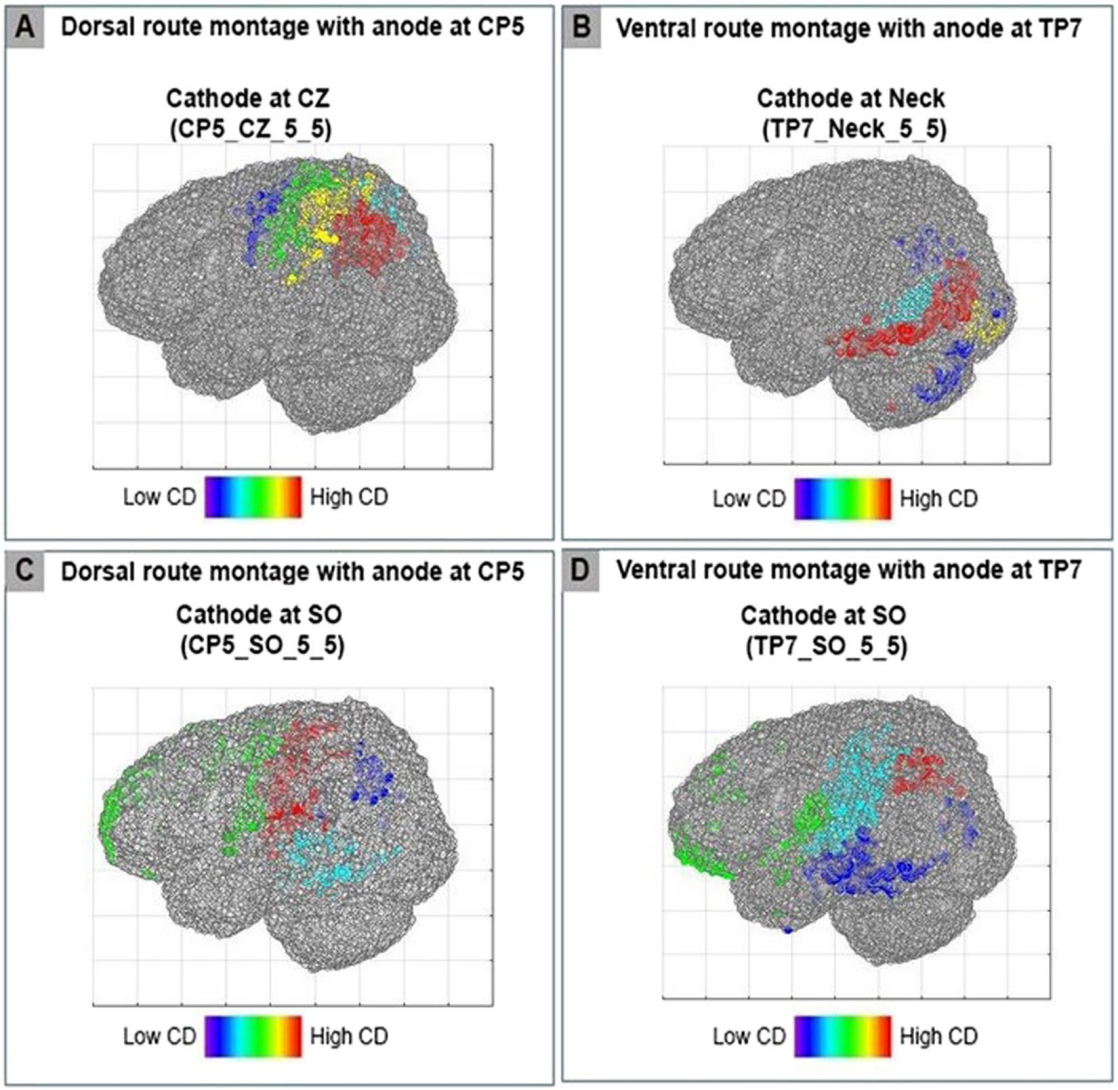
(**A**,**B**) Highest cluster magnitude of current density (CMCD) is seen at supramarginal and middle/inferior temporal gyrus for dorsal pathway montage (CP5_CZ_5_5) and ventral pathway montage (TP7_Neck_5_5), respectively. (**C,D**) Similar clusters are formed for two dorsal and ventral pathway montages (CP5_SO _5_5 and TP7_SO_5_5) where the cathode position is in the same SO position but the anode positions are different at CP5 and TP7.

Cortical regions with high MCD for montages CP5_CZ_5_5 and TP7_Neck_5_5 are represented in Fig. 7A,B, respectively. Decreasing CMCDs values are observed in the following order: supramarginal gyrus, inferior parietal lobe, premotor cortex, motor cortex and superior parietal lobule, for the montage CP5_CZ_5_5. Whereas for the montage TP7_Neck_5_5, decreasing CMCDs values observed in the following order- Inferior temporal gyrus, fusiform gyrus, middle temporal gyrus, lingual gyrus and inferior parietal lobule.

We also found that the clusters of high CMCD values that are formed for two montages (CP5_SO_5_5 and TP7_SO_5_5) with same cathode position at SO but distinct anodal position at CP5 and TP7 are similar to each other. For both montages, the clusters are formed in supramarginal gyrus, inferior parietal lobule, motor cortex, premotor cortex, prefrontal gyrus, superior frontal gyrus, superior temporal gyrus and middle temporal gyrus (shown in Fig. 7C,D).

To supplement the montage selection process further, we additionally displaced anode and cathode from their original positions to 1 cm up and down in the coronal plane and 1 cm left and right in axial plane (supplement). No significant difference in terms of lobe selectivity configuration analysis was found (Fig. S1). However, when we changed the total current intensity from 2 mA to 1 mA, we found a significant decrease in mean MCD per lobe (Fig. S2).

## Discussion

The present study simulated the tDCS montages applied to reading and outlined a computational approach to determine the appropriateness of montage selection. Such customization in montage selection is required in reading and other higher level cognitive functions where multiple neural pathways coexist. A systematic approach that utilises MCD values obtained from the COMETS2/ROAST toolbox^33,34^,^47^ was used to determine three parameters that were based on to select the optimal montages for stimulating, via tDCS, dorsal and ventral pathways of reading, namely the lobe selectivity configuration analysis, number of overlapping coordinates, and cortical regions with high MCDs. These parameters were applied with three principles that guided the optimal choice of the dorsal and ventral montages, required for (1) maximum stimulation at left parietal and temporal lobe for dorsal and ventral pathways of reading with least spread of current to other cortical lobes (2) minimum overlap between the two montages in terms of current spread, and (3) maximum stimulation of the supramarginal gyrus and middle/inferior temporal gyrus in the dorsal and ventral pathways.

For the dorsal pathway, 5 montages (2 conventional and 3 hypothetical) were tested and CP5_CZ _5_5 was found to be optimal. For this montage, the lobe selectivity configuration analysis shows maximum stimulation of left parietal lobe. This was further confirmed when we calculated the cortical area with high MCD and found the highest CMCD at supramarginal gyrus. Similarly, for the ventral pathway, 5 montages (1 conventional and 4 hypothetical) were tested and TP7_Neck_5_5 was found to be the optimal choice. For this montage, the lobe selectivity configuration analysis showed maximum stimulation of left temporal lobe. This was also confirmed by the observation that clusters of highest CMCDs were found in the middle/inferior temporal gyrus. Moreover, this combination of dorsal pathway montage CP5_CZ_5_5 and ventral pathway montage TP7_Neck_5_5 resulted in the least number of overlapping coordinates amongst all the combinations. Therefore, the present analysis suggests two tDCS montages CP5_CZ_5_5 and TP7_Neck_5_5 to be optimal for stimulation of dorsal and ventral pathways of reading, respectively.

The study also analysed the effect of differences in (a) cathode position, and (b) electrode size.

### Effect of cathode position

The present study found that that the maximum CMCDs are found in close proximity to the placement of the anode and spread toward the cathode. This phenomena is expected since the cathode position determines the direction of current flow^7,33,41,49–51^. As a result, we found that when the cathode position is kept constant at SO, and the anode position varies from CP5 to TP7 (i.e. for montages CP5_SO and TP7_SO); the neuroanatomic location of the clusters formed are similar (see Fig. 7C,D). However when cathode positions differs (at CZ and Nape of the Neck), two montages -CP5_CZ and TP7_Neck- generate different localization of CMCDs (Fig. 7A,B). This could be due to the location of the cathode on the scalp in relation to the anode. In the montage CP5_CZ, the relative position of cathode to anode is towards the vertex of the head. The electric potential map (Fig. 2Ai) can also be seen to have the current source at the left hemisphere and the current sink at the vertex of the cerebral cortex. Whereas in the montage TP7_Neck, the relative position of cathode to anode is towards the inion of the head (Fig. 2Biv), reflecting similar location for the current source (i.e., at the left hemisphere) but a different location for the current sink (i.e., at the back of the cerebral cortex). In a situation where two routes coexist and two montages are needed with least amount of overlap in current spread, positioning the cathodes in two different orientations could be an optimal preference.

Moreover, in the bipolar montages, the montages CP5_SO, TP7_SO, and TP7_TP8 appear as two charged poles separated in space (Fig. 5). This was not found for the bipolar montage CP5_CZ and could be due to the short inter-electrode distance resulting in a relatively concentrated spread of current. This is consistent with previous studies that found a significant effect of inter electrode distance on the MCD distribution, with larger inter-electrodes distance producing a relatively more diffuse distribution of current in the brain^28,48,52–54^.

### Effect of electrode size

In 2007, Nitsche and colleagues found that decreasing electrode size results in more focal distribution of current^4^. The present analysis supports these findings. Although the max_MCD increases with a decrease in electrode size from 5 × 5 cm^2^ to 3 × 3 cm^2^, there was no change in the pattern of MCD distribution as indicated by lobe selectivity configuration analysis (see Fig. 3Cii and 3Dii).

Additionally, the sensitivity analysis on each optimal montage CP5_CZ_5_5 and TP7_Neck_5_5 revealed that displacing the electrodes by 1 cm on the scalp had no significant effect on the lobe selectivity configuration analysis (p > 0.05). These findings are consistent with those of Bai *et al*. and Dmochowski *et al*.^48,55^. Lastly, the present study showed a significant decrease (p < 0.01) of average MCD value per lobe for 1 mA compared to 2 mA. Similar findings were also demonstrated experimentally by Iyer *et al*. and Boggio *et al*.^56,57^.

## Limitations and Future Directions

The approach we have reported depends on the MCD distribution obtained as output from COMETS2 for the built-in head model based on FEM. All simulations of complex systems such as MCD distribution are limited by the assumptions inherent to the model.Thus, the validation of computational data with the neurophysiological findings is important. Although it is beyond the scope of the present paper to validate the simulations generated by COMETS2, we note that the results obtained by COMETS2 were concordant with those generated by a second simulation method provided by ROAST. Two studies have found agreement between predictions generated by simulation pipelines like ROAST and electrophysiological measurements: Huang *et al*., demonstrated a strong correlation (r = 0.89) between the predicted electric field and intracortical recordings^58^, and Edward *et al*., reported a correlation of simulated electric field intensities with motor evoked potential measurements^59^.

In addition, our approach has been designed on the MCD output values from COMET2/ROAST without any regard to the direction of the current flow. Accounting for an additional parameter that shows the compliance to directionality will cement the building blocks of systematic approach that we outlined in the present study. However, restricted to simulations, we leave this to future work. We believe that additional experimental work is needed to establish the importance of direction of electric current in clinical practice (especially in reading). It is known that tDCS acts at a subthreshold level (<1 V/m) and can induce both radial and tangential electric field^46^. When the target is in a sulcus, the preferred direction is tangential and if it is on a gyrus, then a radial field may be desirable^46^. Decisions regarding target direction for tDCS experiments will be important when two crucial types of information are available, (i) the exact location of the target region involved in a particular task, and (ii) the direction of electric current (radial/tangential) important to modulate the behaviour (reading in the present case)^46^. Currently, information regarding precise target location and direction important to modulate reading behaviour is not known. When such information is provided by future studies, multi-electrode configuration might be a parsimonious choice because it can restrict the current spread to a particular gyrus or sulcus^46^. In contrast, when such information is not available, sponge electrode configuration, where current is spread over a larger area, is the optimal choice. Moreover, the cortical folding is so extensive that functionally it is not possible to account for current directionality in sponge electrodes. Additionally, Kronberg *et al*. demonstrated that, besides the exogenous current parameter, the endogenous synaptic activity is also important in tDCS^60^. It will be interesting to account for these variations in future studies. Nevertheless, the present analysis presents a useful perspective for selecting an appropriate montage for a commonly used sponge electrode configuration [1 (cathode) × 1 (anode)].

## Conclusion

The present study introduces a computational framework based on obtained MCD (current density) values from COMETS2 toolbox and applies it to identify on optimal pair of tDCS montages for stimulating the two processing routes for reading. We found that in reading, where two pathways coexist in proximity, a montage with anode at CP5 and cathode at CZ could be an optimal choice for stimulating the dorsal pathway. Similarly, a montage with anode at TP7 and cathode at nape of neck could be an optimal choice when the ventral pathway of reading needs to be stimulated. The analysis also showed that amount of MCD to the target area increases with decrease in electrode size but that there is no change in the pattern of current distribution. Therefore, our findings suggest an electrode size of 5 × 5 cm^2^ consistent with prior reading studies using that electrode size^15,16^. This framework is shown to be especially useful as it allows simultaneous evaluation of multiple montages, reducing ambiguity about montage selection.

## Supplementary information

SUPPLEMENTARY
