# Supplementary material for: Simulation Analyses of tDCS Montages for the Investigation of Dorsal and Ventral Pathways
Source: Sci Rep. 2019 Aug 21;9:12178. doi: 10.1038/s41598-019-47654-y (PMC6704077; doi:10.1038/s41598-019-47654-y)
Supplement: Supplementary file 1 — SUPPLEMENTARY [file 41598_2019_47654_MOESM1_ESM.docx]

**Supplementary**

**Simulation Analyses of tDCS Montages for the**

**Investigation of Dorsal and Ventral Pathways**

Sagarika Bhattacharjee^1^, Rajan Kashyap^2^, Brenda Rapp^3^, Kenichi Oishi^4^, John E. Desmond^5^, SH Annabel Chen^1, 2, 7^

^1^ Psychology, School of Social Sciences, Nanyang Technological University, Singapore,

^2^ Centre for Research and Development in Learning (CRADLE), Nanyang Technological University, Singapore

^3^The Johns Hopkins University, Kreiger School of Arts and Sciences, Baltimore, United States,

^4^The Johns Hopkins University, School of Medicine, Baltimore, United States,

^5^Department of Neurology, The Johns Hopkins University, School of Medicine, Baltimore, United States,

^7^Lee Kong Chian School of Medicine (LKC Medicine), Nanyang Technological University, Singapore

__________________________________________________________________________

# Sensitivity analyses on selected montages

On the two decided montages some additional analyses were performed.

1. The effect of displacement of electrodes over the scalp was tested because of likelihood in variation during manual electrode fitting procedure. For this, the anode position was displaced 1 cm up and down in the coronal plane and 1 cm left and right in axial plane, maintaining the same the cathode position. These displaced montages were simulated and average CD per lobe were calculated for each montage. The mean CD values of each lobe were compared between the original and displaced montages for test of significance by one way ANOVA
2. Similar to the above analysis, the effect of change in total current intensity for 2mA, 1.5mA and 1mA were tested for significant difference over average CD per lobe.


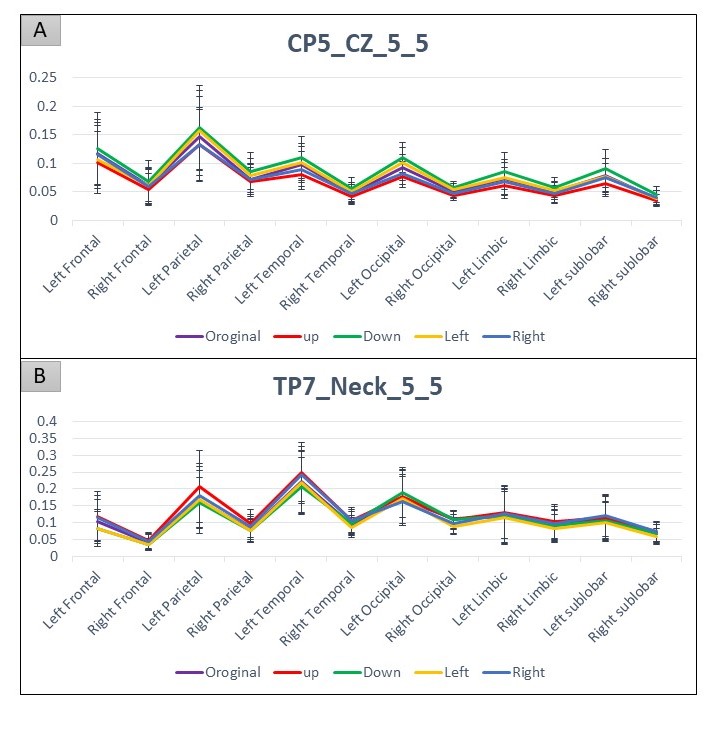


Fig 1S (A) and (B) compares the mean CDs in each cortical lobe for displacements in the scalp in dorsal (CP5_CZ_5_5) and ventral pathway montage (TP7_Neck_5_5), respectively. The original position (violet) of anode is compared with displacement of cathode by 1cm up (red) and 1cm down (green) in coronal plane, 1cm left (yellow) and 1 cm right (blue) in axial plane. There is no significant (*P* < .05) difference in the mean CD value for each cortical lobe with displacement of anode.


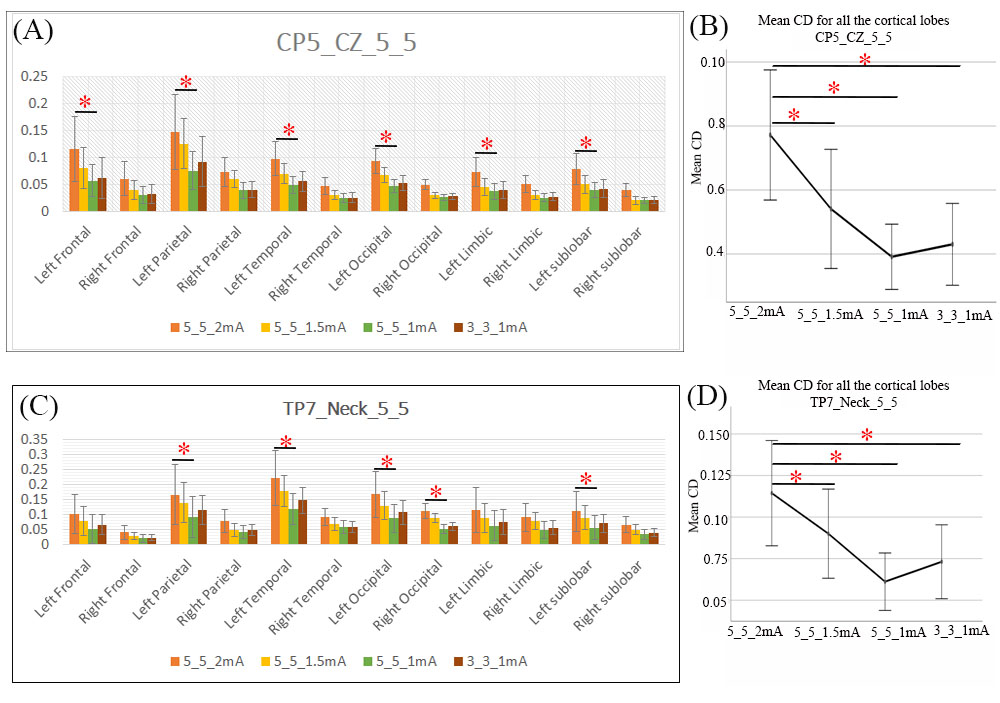


Fig 2S (A) compares the effect of change in current intensity from 2mA (orange), 1.5 mA (yellow), and 1 mA (green) for dorsal pathway montage CP5_CZ_5_5. (B) There is significant difference in mean CD for all the cortical lobe with change in current intensity; F= 57.89, (*P* < 0.05). This also demonstrates that mean CD reduction at 1mA, 1.5mA relative to 2mA demonstrates a linear relationship. Post- hoc analysis shows the significant difference is seen in left frontal, left parietal, left temporal, left occipital, left limbic and left sublobar lobes. Moreover, when CP5_CZ_5_5 with 2mA (orange) is compared with CP5_CZ_3_3 with 1mA (brown), there is a significant difference in average CD per lobe (*P* < .05). This suggests the decrease in CD by decreasing the current intensity cannot be compensated by decreasing the electrode size to 3 × 3 cm ^2^. This was tested because the analysis in main manuscript showed amount of mean CD per lobe might increase with decrease in electrode size. (C) and (D) shows the similar result as (A) and (B) for the ventral pathway montage TP7_Neck_5_5.
